# Barbadoes Aloes Not in the Market

**Published:** 1898-09

**Authors:** 


Barbadoes Aloes Not in the Market. The *Myer Brothers' Druggist* states, upon reliable authority, that true Barbadoes aloes is not an article of commerce, none having been permitted to be exported for the last ten years. What now sells as such is a product of Curacoa, which closely resembles the genuine.—*Western Druggist.*

Pure Air. Mountain-climbing scientists have ascertained that but few microbes are found at an altitude of 2000 feet, and absolutely none are found at an elevation of 5000 feet.

